# Suicidality Among Syrian Refugee Children in Jordan

**DOI:** 10.1007/s10597-023-01160-8

**Published:** 2023-07-22

**Authors:** Rebecca Dehnel, Heyam Dalky, Subashini Sudarsan, Wael K. Al-Delaimy

**Affiliations:** 1 Long Beach Memorial Family Medicine, Long Beach, USA; 2 Jordan University of Science and Technology, Irbid, Jordan; 3 UC San Diego, San Diego, USA; 4 UC San Diego Division of Global Health, Department of Family and Preventative Medicine, San Diego, USA

**Keywords:** Syrian, Refugee, Children, Depression, Suicidality, Adverse childhood event (ACE)

## Abstract

History of adverse events in childhood is one of the strongest predictors of developing negative mental health outcomes with suicidality being the most devastating consequence. Syrian refugee children are at very high risk of developing mental illness, however, the prevalence and significance of suicidal thoughts among this population remains undocumented. A total of 339 Syrian refugee children and adolescents aged 10 to 17 years and their parents living in Jordan were surveyed to assess resilience, depression and suicidality. Demographics and parental depression were correlated with child suicidality. Multivariate logistic regression analyses were used to determine the predictors of suicidality within this population. A total of 94 (27.7%) children reported suicidal statements. In the multivariate analyses we found that younger children were more likely to state suicidal ideation than older children. Of the children who stated suicidal ideation, 49 were in primary school, 19 in pre-secondary and 26 in upper-secondary school. In the multivariate analyses, mild (OR 2.633 (95% CI 1.283, 5.709)) and high (OR 6.987 (95% CI 3.532, 14.614)) depression levels among the surveyed children were predictive of suicidality. Experiencing bullying was also predictive of suicidality (OR 2.181 (95% CI 1.179, 4.035)) when compared to those who did not report any bullying. We report high rates of suicidal ideation among Syrian refugee children, especially in children with exposure to pre-existing depression or bullying. Prevention through raising awareness, education and early detection of depression are needed to address suicidality in this and other vulnerable populations of children.

## Introduction

It has been over a decade since the start of the Syrian Civil War and the resulting Syrian refugee crisis. Since 2011, over 6.6 million Syrians have fled Syria and an additional 6.7 million remain internally displaced (UNHCR, 2022). It is estimated that half of the millions who are displaced are children under the age of 18 and 40% are under the age of 12 (UNICEF, 2022). By virtue of the refugee reality, refugee children have very high exposure rates to adverse childhood events (Felitti et al., 1998). Exposure to situations of violence, poverty, lack of stability and loss of family members to separation, imprisonment and death are common (Opaas & Varvin, 2015) and force refugee children to grow up and develop in crisis (Montgomery, 2010). Refugee children do show incredible protective resilience (Pieloch et al., 2016), however many still go on to develop negative mental health outcomes such as depression, anxiety and post-traumatic stress disorder (Fazel & Stein, 2002; Lustig et al., 2004; Reavell & Fazil, 2017). Feelings of insecurity, sadness, fear, isolation and worthlessness have been stated by refugee children across multiple contexts which raises significant concern for suicidality risk.

Even outside of the Syrian context there has been sparse research on the topic of suicidality among refugees. This is likely attributable to lack of reliable data, under-reporting of suicidal behavior and difficulty obtaining permission to study suicidality given its politically sensitive nature and high degree of stigmatization (Vijayakumar, 2016). Of the available studies, the incidence of suicidality among refugees appears to vary greatly, estimated at 3.4 to 34% according to a 2010 review (Vijayakumar & Jotheeswaran, 2010) and even as high as 91% in a study of Afghan refugee women (Rahman & Hafeez, 2003). Since that review in 2010, suicidality rates continue to vary greatly between studies (Nam et al., 2021; Norredam et al., 2013; NPR, 2017; Rahman & Hafeez, 2003; Tekeli-Yesil et al., 2018; UNHCR, 2021). Most research has focused on small groups of specific refugee communities within specific host countries (Nam et al., 2021; Norredam et al., 2013; NPR, 2017; Rahman & Hafeez, 2003; Tekeli-Yesil et al., 2018; UNHCR, 2021; Vijayakumar & Jotheeswaran, 2010). In Denmark, a large population-based study reported suicide rates being lower among male refugees than males in the general population, but there was no difference among women (Norredam et al., 2013). Recently, a study of North Korean refugee women living in South Korea found that a very high percentage (46%) reported suicidal ideation (Nam et al., 2021). Such incongruence highlights how nuanced the topic of suicidality is and demonstrates the need for studies of individual refugee communities to better understand the prevalence of suicidality within each of them in order to create interventions that will have a positive impact.

Within Syrian refugee populations there has been rising concern surrounding the impact of psychological trauma and resulting suicidal ideation. Individual interviews and oral reports indicated for years that the suicide rate among Syrian refugees was high (NPR, 2017; UNHCR, 2021) and in 2017 Al Ibraheem et al. reported that 11.3% of Syrian participants in their study stated that they had a plan or history of attempting suicide, a rate which is over two times greater than that in the United States of America (Al Ibraheem et al., 2017). These findings were followed later in 2018 by Tekeli-Yesil et al. who reported that more than 50% of their sample were at high risk for suicide (Tekeli-Yesil et al., 2018). Research on Syrian refugee suicidal ideation in adults has been sparse and there have been no research studies focused on Syrian refugee children. However, there are significant anecdotal accounts of suicide attempts within refugee camps, most recently focused on reports of Syrian refugee children dying by suicide (Aljazeera, 2020; Info Migrants, 2021).

In 2018 a series of cases were reported of Syrian refugee children as young as ten years old attempting suicide within refugee camps in Greece (BBC, 2018; Médecins Sans Frontières, 2018) followed in 2019 by reports of children found banging their heads against walls and other children as young as seven stating that they wanted to die (BBC, 2019a). Within that same year, the cases of Syrian refugee children committing suicide after resettlement made international headlines. Most notably a nine-year-old girl who had experienced extreme bullying after resettling in Canada (Global News, 2019) and a nine-year-old boy who had been attacked by classmates in Turkey (BBC, 2019b). In 2020 suicide rates in Northern Syria reached a record high, and an estimated one in five of all recorded suicide attempts and deaths were children (Save The Children, 2021) with additional published reports of Syrian children committing suicide after being denied resettlement (Info Migrants, 2020).

We surveyed a cohort of Syrian refugee parents and their children living in Jordan in 2018 to better understand the prevalence of trauma, mental health challenges and resilience among Syrian families. The aim of this analyses was to understand mental health patterns and life experiences of Syrian refugee children that may predispose them to suicidality.

## Method

### Participants and Procedure

Participants for the study (N = 339) were Syrian refugee children and adolescents and their primary guardian living in Jordan. The study protocol and methodology has been described in an earlier publication (Dehnel et al., 2021). In brief, families were approached for participation in the period of July to October 2018 at a major community clinic for refugees in Ramtha, Northern Jordan, in addition to numerous Syrian community centers in the major nearby city of Irbid. Families were also contacted through community-based organizations in an attempt to create a more diverse sample. Eligible participants were enrolled with a primary guardian if they were Syrian refugees aged 10 to 17. Due to illiteracy among the study population, the majority of surveys were completed verbally in private areas to maintain confidentiality. Research protocol was reviewed and approved by relevant institutional review boards and all study participants completed the informed consent in Arabic verbally due to the sensitive nature of the research questions and the importance of confidentiality in this setting. All children who expressed any form of suicidal ideation were immediately referred and followed-up by clinic personnel per clinic protocol.

### Instruments

Instruments included the Children’s Depression Inventory 2: (CDI-2), the Hopkins Symptom Checklist (HSCL-25), the Strengths and Difficulties Questionnaire (SDQ). These surveys were selected as they had been previously translated into Arabic and validated in previous studies or were provided in Arabic by the tool developer (East et al., 2018; Panter-Brick et al., 2018; Yun et al., 2021).

#### Demographics

A demographics questionnaire developed for this project assessed basic demographic information including age, gender, ethnicity, and primary guardian role (see Table 1).

**Table 1:** Participant demographics and variable descriptives

Demographics	
Age in years	
Mean (SD)	13.4 (2.3)
Median (Range)	13 (10–17)
Gender, n (%)	
Male	85 (25.1)
Female	252 (74.3)
Unreported	2 (0.01)
Ethnicity, n (%)	
Arab	335 (98.8)
Other	1 (0.003)
Unknown	3 (0.01)
Guardian completing form, n (%)	
Mother	315 (91.8)
Father	18 (5.2)
Grandmother	4 (1.2)
Grandfather	2 (0.6)
Other	4 (1.2)
Psychosocial variables	
Child depression (CDI-2)	
Mean (SD)	14.4 (7.8)
Median (Range)	14 (0–40)
Depression score higher than median, n (%)	173 (51.0)
Depression score lower than median, n (%)	166 (49.0)
Child suicidality (CDI-2: item 8)	
“I do not think about killing myself”, n (%)	239 (70.5)
“I think about killing myself but would not do it”, n (%)	81 (23.8)
“I want to kill myself”, n (%)	13 (4)
Did not answer, n (%)	6 (1.7)

#### Child Depression

Depressive symptomatology in children was assessed using the Children’s Depression Inventory 2: (CDI-2) (Kovac, 2010). This survey is a brief self-report test that assesses cognitive, affective and behavioral signs of depression in children and adolescents. The CDI-2 contains 28 items, each of which consist of three statements. For each item, the individual selects the statement that best describes their feelings. For example, “I do not feel alone”, “I feel alone many times”, or “I feel alone all the time”. Individuals with higher scores on the CDI-2 are characterized as having higher rates of depression. The initial version of the scale was first adapted for use with Arabic speakers in 2006 with an overall alpha reliability coefficient of 0.85 (Al-Balhan, 2006). Multiple cut off scores for significant depression using this measure have been theorized in the literature for various populations. For example, a score of 13 and above suggests a high likelihood of clinical depression (Kovacs, 1992), while a score of 16 and above indicates a level of depressive symptoms that impacts children’s everyday activities with family, friends and at school (Roelofs et al., 2010).

#### Parent Depression

Depression in parents was assessed using the Hopkins Symptom Checklist (HSCL-25) which is derived from the 90-item Symptom Checklist (SCL-90) (Derogatis et al., 2022) and is used as a screening tool for symptoms of both depression and anxiety. The first 10 items of the scale measure levels of anxiety while the last 15-item measure depression. Each question is scored as 1 (not at all) to 4 (extremely). Participants answer based on their feelings over the past month. Higher total scores on the last 15 items (HSCL-D) correspond with higher depression.

#### Suicidality

Suicidality was assessed using the Children’s Depression Inventory 2 (CDI-2) question regarding suicidality (Item 8) (Kovacs, 1992). Participants are asked to characterize the degree to which they have had suicidal thoughts over the past two weeks. For example, (1) “I do not think about killing myself”; (2) “I think about killing myself, but would not do it”; and (3) “I want to kill myself”. For the purpose of the current analysis, marking option 2 or 3 was coded as positive for suicidal ideation. Suicidality is derived from the CDI score and hence for meeting statistical conditions, the CDI score was calculated without the suicidality measure.

#### Strengths and Difficulties

The Strengths and Difficulties Questionnaire (SDQ) was used to assess children and adolescent behavior, emotions and relationships from the perspective of their primary guardian and has demonstrated adequate discriminant and predictive validity (Goodman et al., 1997). The SDQ contains 25 items and five clinical scales: hyperactivity/inattention, emotional symptoms, conduct problems, peer relationship problems and prosocial behavior. For each clinical scale, the score can range from 0 to 3, with 0 (not true), 1 (somewhat true), and 2 (certainly true). Higher scores on the prosocial behavior subscale reflect strengths, whereas higher scores on the other four subscales reflect difficulties within each category. The measure is completed by either a primary guardian, teacher or participant. For the purposes of this study only the parents were asked to complete the SDQ. Question 19 from the SDQ (SDQ19) asks if the child was “Picked on or bullied by other youth” during the last 6 months or recent school year and was used as a measure to determine whether a child had experienced bullying from the parent’s point of view. Children of those parents that answered either 1 or 2 for the question were considered as ‘Perceived as being bullied’ and children of those parents that answered 0 to the question were considered as ‘Not perceived as being bullied’.

## Analysis

### Statistical Methodology

Descriptive statistics were run for means, medians and ranges of scores of child and parent measures. All variables were used as continuous variables in the regression analyses. The median value was used as a cut-off point to generate categorical variables of depression since defined cut-off scores for these measures are not established and are highly debated within the literature.

Correlation analysis was performed between suicidality and multiple variables, including child depression (CDI without CDI Item 8) score, parent’s depression score (HSCL-25), gender of the child, age of the child, child’s education status, family income and child bullying factor (SDQ19). Only those variables that had a significant correlation with suicidality were included in the multivariate regression analysis.

The SDQ19 variable was used as a categorical variable in the multivariate logistic regression and as a discrete variable in assessing the correlation between bullying and child suicidality.

The parent depression score was calculated based on an H score. Parent’s depression score was used as a continuous variable in assessing the correlation between parent depression and child suicidality whereas in the multivariate logistic regression, the variable was used as a categorical variable. The score was categorized based on the tertile values.

### Multivariate Logistic Regression Analysis

The variables that show slightly stronger associations with child suicidality were put in a model with Child Suicidality as the response variable and Child Depression, Parent Depression, Child’s Age, Perceived Bullying of the Child as explanatory or predictor variables. Child Suicidality was used as a categorical variable and hence logistic regression was used.

### Explanatory Variables/Predictor Variables

Child Depression was used as a categorical variable and the variable was categorized based on the tertile values. There were three levels: No Depression, Mildly Depressed and Highly Depressed. The level used as reference was No Depression. The Parent’s Depression was also used as a categorical variable and the variable was divided into three levels using the tertile values. No Depression was used as the reference level. Child’s Age was used as a continuous variable.

Bullying was used as a categorical variable and those that answered either 1 or 2 were categorized as parents that felt their child was bullied and those that answered 0 were categorized as those parents that did not feel their child was bullied.

### Effect Modification

Age, Gender, and Parent’s Depression were used in the effect modification analysis. For Age, the study participants were divided into two groups, those that were between 10 to 12 and those that were between 13 and 17. The former group was considered “Children” and the latter group was considered “Teens”.

For the Parent Depression variable, subjects were divided based on the tertile score of the Parent Depression scale. Thus, three groups were formed, children whose parents had mild depression (score 14–24), children whose parents had moderate depression (score 25–32) and those children whose parents had high depression (score 33–53). In each group, Child Suicidality was regressed upon Child Depression score and Bullying to see if Age, Gender and Parent Depression modified the effect of Child Depression and Bullying on Child Suicidality.

### Statistical Software

All analysis was performed using the R statistical package (Version: Rstudio 1.4.156).

## Results

### Prevalence of Suicidality Among the Study Population

Of 339 children, 81 children selected “I think about killing myself but would not do it” and 13 children selected “I want to kill myself”, for a combined total of 94 (27.7%) children. Six children did not mark an answer to the question. Among the 94 children, 70 (74%) were girls and 24 (26%) were boys. Suicidality was seen more among 53 children younger than 13 years with and 50 children being teens. A total of 49 children in primary school showed suicidal ideation compared to 19 in pre-secondary and 26 in upper secondary. Distribution of suicidality based on age, gender and education level can be seen in Table 2. Of 333 parents, 72 (21.6%) stated that their child was being bullied, whereas 261 (78.3%) parents did not. The parents of the six children who did not answer the suicidal ideation question were excluded from frequency analysis of perceived bullying. The correlation between parental depression and child suicidality was a weak, but significant correlation (r = 0.2, p = 0.0004).

**Table 2:** Child suicidality based on multiple variables

a. Distribution by gender		
Child suicidality	Gender	
	Male	Female
Yes	24	70
No	60	177

b. Distribution by age								
Child suicidality	Age of child (years)							
	10	11	12	13	14	15	16	17
Yes	17	12	24	13	6	10	12	9
No	19	50	15	31	26	20	42	27

c. Distribution by education level					
Child suicidality	Education level				
	No education	Pre-secondary	Primary	Upper secondary	Missing
Yes	0	19	49	26	6
No	2	57	110	67	

There was a moderate and significant correlation between child suicidality and child depression with a correlation coefficient of 0.4 (p < 0.0001) (Table 3). The correlation coefficient between child suicidality and bullying was a weak, but significant association of 0.2 p = 0.0003. Correlation between emotional SDQ and child suicidality was also weak at 0.2 (p = 0.001). There was no correlation between child age and child suicidality (0.033, p = 0.5). In the Multivariate Logistic Regression Analysis, child depression and bullying were found to be the most strongly associated with suicidal thoughts in children in this sample (Table 3).

**Table 3:** Multivariate logistic regression analysis

Predictor variables	Estimate	Odds ratio	95% CI lower OR	95% CI upper OR	p value
Child depression—low	0.968	2.633	1.283	5.709	0.01
Child depression—high	1.944	6.987	3.532	14.614	< 0.0001
Parent depression—low	0.039	1.04	0.513	2.117	0.91
Parent depression—high	0.647	1.91	0.979	3.792	0.06
Bullying	0.78	2.181	1.179	4.035	0.012
Child age	− 0.12	0.887	0.788	0.994	0.04

#### Child Age

The age of the child was found to be strongly, but inversely associated with child suicidality. Thus as the age of child increases by 1 year, the odds the child would state suicidality decreases by 11.3% (OR 0.887 95% CI 0.788, 0.994).

#### Child Depression

The odds that a child who was categorized as mildly depressed and also stating suicidal ideation was 2.633 (95% CI 1.283, 5.709) compared to a child who was categorized as not depressed. The odds that a child who was categorized as highly depressed also stating suicidal ideation was very high at 6.987 (95% CI 3.532, 14.614) compared to a child who was categorized as not depressed.

#### Parental Depression

Neither low nor high depression levels in parents were found to be associated with child suicidality. The p-values of the association of child suicidality with low level of parent depression and high level of parent depression were 0.91 and 0.06 respectively.

#### Bullying

The odds that a child who was perceived as experiencing bullying would state suicidality was 2.181 (95% CI 1.179, 4.035) times more than the child who was not perceived as being bullied.

### Effect Modification

#### Age

**Fig. 1:**
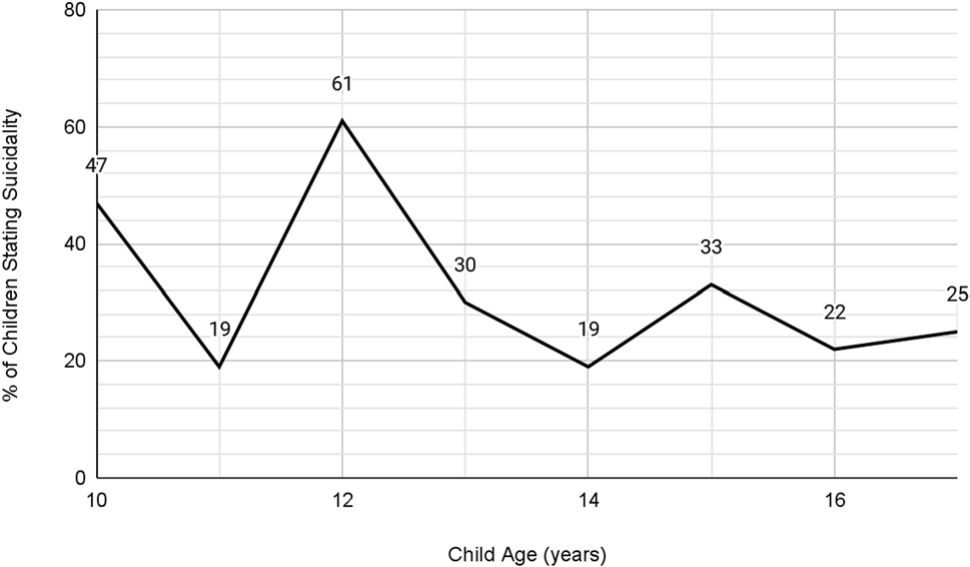
Percentage of children stating suicidal ideation by age

Age might be an effect modifier and in order to test this the children in the study were divided into two groups. Those between age 10 and 12 formed a ‘Children’ group and those between age 13 and 17 formed the ‘Teen’ group. In each group, regression analyses was performed with child suicidality as the response variable and child depression, parent depression and bullying as explanatory variables. In the 10–12 year old group, bullying (OR 3.95, 95% CI 1.68, 9.53) and child depression (OR 1.07, 95% CI 1.02, 1.13) were associated with child suicidality. In the teens group, only child depression (OR 1.21, 95% CI 1.14, 1.31) was found to be strongly associated with child suicidality. Percentage of children stating suicidal ideation by age can be seen in Fig. 1.

#### Gender

To see if gender was an effect modifier, the study sample was divided based on gender. In each group, child depression, age of the child, bullying and parent depression were used as explanatory variables and child suicidality was used as the dependent variable. In male participants, only child depression was found to be associated with child suicidality (OR 1.08, 95% CI 1.00, 1.16). Whereas in female participants both child depression (1.16, 95% CI 1.10, 1.22) and bullying (3.11 95% CI 1.43, 6.83) were found to be associated with child suicidality. Since there is a difference in the effect of bullying on child suicidality between the two genders, gender can be considered as an effect modifier.

#### Parent Depression

Children in the study group were divided based on their parent’s depression scores into three groups: children whose parents had mild depression, moderate depression, or high depression. In all three groups, child depression was associated with child suicidality. Thus, no effect modification was observed but in the case of bullying those children whose parent had high depression showed stronger association (OR 2.79, 95% CI 1.19, 6.74) with child suicidality.

## Discussion

Now over a decade later, the Syrian refugee crisis continues to carry an economic and psychological impact that will last for generations. High rates of depression and anxiety among refugee parents are being reflected in their children with a growing concern that children also bear a strong emotional burden from displacement. Suicidality is a tragic and preventable outcome of this psychological stress, but the prevalence of suicidal thoughts and behaviors among refugee children is poorly understood, especially in Muslim communities where stigma has a strong influence (Gearing & Lizardi, 2009). To our surprise among the 339 Syrian refugee children we interviewed, nearly a third endorsed suicidality ideation. Of those children thirteen openly stated that they wanted to end their lives. To the best of our knowledge, this has not been reported previously within this population.

Suicidal thoughts and behaviors during childhood are seen as relatively rare events with a prevalence that increases significantly during adolescence (Dervic et al., 2008; Nock et al., 2012). Contrary to this, we found within our cohort that younger children were more likely to state suicidal thoughts than older children. An explanation for this finding could be that younger children within this population have not yet learned the full extent to which suicidality is viewed as a forbidden subject and were more likely to be honest about their thoughts during interviews. It also could reflect the high level of trauma those children are experiencing. A counter argument could be that the questions being posed were misunderstood by younger participants causing them to incorrectly select that they were having suicidal thoughts or wanting to end their lives. This is a valid concern given that most surveys are standardized based on children who have not experienced the extreme educational and social disruption that refugee status places on children. However, consistent with prior studies, the prevalence of depression among this cohort of children was very high and correlated with suicidality. This validates that children were choosing their answers with an understanding of the meaning of suicidality.

Higher rates of depression among Syrian refugee children have been repeatedly documented (Fazel & Stein, 2002; Lustig et al., 2004) and are a reflection of the hardship and emotional turmoil that the refugee experience embodies. The relationship between depression and suicide is well-established with depression being highly associated with suicidality among adults and children (Coryell & Young, 2005). However, it is unclear which other factors may influence suicidality within refugee children. Given the crucial influence that parents have in children’s lives, we hypothesized that the children who stated suicidal thoughts would be more likely to have parents with higher depression scores and more significant trauma histories, however, we found no association between these factors. This could be attributed to the fact that in this highly traumatized population there are factors such as social isolation, bullying, discrimination, recent loss or separation from family and friends, fear of detainment or death, inhumane living conditions, low income status, and lack of access to education and healthcare that may play a more influential role on suicidal ideation of children than the potential transgenerational impact of their parents' mental health.

One of these external factors may be related to children’s social experiences both during their transition out of their home countries and into new communities during resettlement. Children of parents who stated that their child had experienced bullying had higher rates of suicidality. This is consistent with multiple systematic reviews which support that bullying interferes with normal developmental and educational success of children (Armitage, 2021; Vanderbilt & Augustyn, 2010) and also places children at risk for suicidal thoughts and behaviors (Kim & Leventhal, 2008). This is particularly pertinent for refugee populations given the ostracism that many children face when they enter new communities, especially educational settings with cultural barriers. Syrian refugee children have to manage with national identity and dialect differences compared to their Jordanian counterparts. Interestingly, in 2015 Lim et al. found that while refugee children did not report higher rates of bullying than their peers, bullying was more likely to be experienced by younger refugee children than initially thought (Lim & Hoot, 2015). In 2020 Samara et al. found that younger refugee children reported more peer problems and functional impairment compared to older refugee children and control groups and that experiences of bullying appeared to account for this difference (Samara et al., 2020). While extensive further work in this field is still needed, these preliminary studies raise concern that highly traumatized refugee children are experiencing bullying at earlier ages which may predispose them to higher risk for suicidal thoughts and behaviors.

### Limitations

These findings should be interpreted with the following limitations in mind. Suicidal thoughts were assessed using one question on the CDI-2. Future studies should use a more expansive assessment of suicidal thoughts in addition to characterizing history of past suicidal behaviors to further explore the factors that influence suicidality among this population. Bullying was assessed using a limited survey method as well with only parents providing input on whether the child had experienced bullying. Given that parents historically under-report bullying and that rates of bullying are more accurate when both child and adult input is provided (Casper et al., 2015) future studies should also assess bullying experience from multiple perspectives.

For the measures that children did complete we were dependent on their interpretation of the survey questions regarding depression and suicidality symptoms. This subjective response might underestimate the true associations found as there was no pediatric psychiatrist to formally assess clinical depression or suicidality. However, most studies have relied on these measures for children and the instruments related to depression have been validated previously. Lastly, the majority of children in this sample were female which may have had an unintended influence on the results given higher rates of suicidal thoughts among girls (Roland, 2002). Despite this, it is interesting that bullying was still significantly correlated with suicidality given that historically boys tend to experience bullying more so than girls (Pellegrini & Bartini, 2000; Vaillancourt et al., 2008). This might speak to higher rates of bullying for Syrian refugee girls than prior population studies have suggested and should be explored further.

## Conclusions

The refugee experience remains a reality for millions globally with continued international conflict resulting in countless additional children meeting refugee status every year. The mental health impacts of being a refugee as a child are becoming better documented and understood, however the consequence of this impact is still a developing area of study. Understanding suicidality within the Syrian refugee context is crucially important to protect children from preventable death and their families from additional tragedy. Our results indicate a major mental health problem among this population that may be preventable.

Ideally, future interventions should have a multilevel approach with efforts directed at home, school, and community settings. A focus on parent education concerning suicidality could lessen stigma and create opportunities for children to discuss these issues directly with their parents and create prevention opportunities. School-based interventions could make teachers and staff more aware of these issues and could lead schools to prioritize decreasing bullying, especially for refugee children in resettled and transitional countries, as this was one of strongest predictors of suicidality risk in children in our study. Additionally, community-based interventions could focus on screening for suicidality among refugee children in partnership with resettlement agencies. These community-focused organizations are uniquely positioned to have developed positive rapport with families and may be able to create a space of trust for children to disclose thoughts and history of suicidal ideation.

The prevalence and severity of suicidality among Syrian refugee children is slowly becoming better understood. However, only through appropriate screening can interventions and follow-up be done to keep this highly vulnerable population of children safe from self-harm.
